# Supplementary material for: Viral mapping in COVID-19 deceased in the Augsburg autopsy series of the first wave: A multiorgan and multimethodological approach
Source: PLoS One. 2021 Jul 19;16(7):e0254872. doi: 10.1371/journal.pone.0254872 (PMC8289110; doi:10.1371/journal.pone.0254872)
Supplement: S1 File — (PDF) [file pone.0254872.s001.pdf]

IHC SARS, anti Sars 0.48mg/ml 4F3 from Reiche Laboratory, Leipzig, Germany

- deparaffinize
- 3min Aquadest
- 10min 3% H_2O_2 prepared in Aquadest
- 3 min Aquadest
- Unmasking: citrate buffer ph 6.00 MW 20min 700W
- Transfer to coverplates
- 3x TBS wash
- Block: Goat normal serum, 100% 30 min RT
- Primary Antibody: Anti Sars 4F3 0.48mg/ml 1:200 üN 4°C
- 3x TBS wash
- Secondary antibody Anti mouse biot. 30min RT
- 3x TBS wash
- BC Reagent 200µl 30min RT
- 3x TBS wash
- AEC 10min wet chamber
- Aquastop 2x
- Hämatoxilin: 2min
- bluing in cold tap water for approx. 10min.
- Aquadest every 5min
- cover with Aquatex

Reagents

Anti Mouse IgG (H+L) Biotinylated, Vector Lab

ABC Kit Vectastain Elite   PK 6100

AEC Substrat Chromogen Ready to use K3464 11092977
