# Supplementary material for: Viral mapping in COVID-19 deceased in the Augsburg autopsy series of the first wave: A multiorgan and multimethodological approach
Source: PLoS One. 2021 Jul 19;16(7):e0254872. doi: 10.1371/journal.pone.0254872 (PMC8289110; doi:10.1371/journal.pone.0254872)
Supplement: S2 File — (PDF) [file pone.0254872.s002.pdf]

RNAscope® 2.5 RED Detection & IHC

FFPE cuts & dewaxing

Materials: TOMO Slides, 2 x 200 ml Xylene, 2 x 200 ml 100% Alcohol.

1. Cut the Kerosene blocks into 2 µm thick, put them into a water bath (at 40 - 45°C), place them on the TOMO slides and let them dry overnight at RT.
2. Bake the next day for 1 hour at 60°C.
3. Place in xylene for 5 minutes at RT.
4. 5 minutes in Xylene at RT.
5. 1 minute in 100% Alcohol.
6. 1 minute in 100% Alcohol.
7. Air-dry for 5 minutes at RT.

RNAscope® Hydrogen Peroxide, H_2O_2

Materials: RNAscope® Hydrogen Peroxide, 200 ml 1X RNAscope® Target Retrieval Reagents (diluted to 1:9, 180 ml diH_2O with 20 ml 10X RNAscope® Target Retrieval Reagents mix well), dH_2O, Steamer, digital thermometer.

1. Switch on the steam cooker. Place a beaker with 200 ml 1X RNAscope® Target Retrieval reagents and another beaker with diH_2O.
2. Apply 5 to 8 drops of RNAscope® Hydrogen Peroxide.
3. Incubate for 10 minutes at RT.
4. Rinse up and down 2 x 3-5 times with dH_2O.
5. place in diH_2O for 10 seconds at 80°C to acclimatize and then place in 1X RNAscope® Target Retrieval Reagents for a further 15 minutes (temperature between 102 - 104°C). Digestion time varies depending on tissue type, see table - last page.
6. Rinse with diH_2O for 15 seconds.
7. Place in 100% alcohol for 3 minutes.
8. Air dry at RT.
9. Draw barrier

RNAscope® Protease Plus & Staining with Fast RED

Materials: HybEz Oven, HybEz Slide Rack, HybEZ™ Control Tray, RNAscope® Protease Plus, Target Probes (Hs - PPIB, Dab-β, V-nCoV20019-s: ssRNA(+), V-nCoV20019-s-Sense: ssRNA(-), warm up for 10 minutes at 40°C in a water bath before use), AMP 1-6 Reagents, 3 liters 1x Wash Buffer (diluted 1: 49 mix 2940 ml diH_2O with 50x RNAscope® Wash Buffer, warm up the Wash Buffer for 10 minutes at 40°C before use), Fast RED A-B (diluted 1:60), 200 ml counterstaining reagent (diluted 1: 1, mix 100 ml Hematoxylin Gill's I with 100 ml dH_2O), 0.02% ammonium hydroxide solution (mix 1.43 ml 1N ammonium hydroxide with 250 ml dH_2O), 200 ml xylene, Gabis hot plate, Eukitt™ Cover media, 24 mm x 50 mm cover glasses.

- Preheat 1st oven to 40°C.
- Add 5 drops of RNAscope® Protease Plus on each cut.
- 3.Place the HybEZ™ rack in the oven for 30 minutes at 40°C (The digestion time varies according to tissue type, see table - last page).
- Pour away excess liquid.

- Rinse 3 - 5 times in diH_2O
- Add 4 drops from the target probe.
- Place in the oven for 2 hours at 40°C
- Pour away excess liquid.
- Rinse 2 times with 1X Wash Buffer for 2 minutes at RT.
- Add 4 drops from the AMP 1.
- Place in oven for 30 minutes at 40°C.
- For hybridization of reagents AMP 2 - 6 repeat steps 8 to 11! **PAY ATTENTION TO THE TIMES AND TEMPERATURES OF DIGESTION!**

**Reagents**	**Time**	**Temperatures °C**
AMP 2	15 Minutes	40°C
AMP 3	30 Minutes	40°C
AMP 4	15 Minutes	40°C
AMP 5	30 Minutes	RT
AMP 6	15 Minutes	RT

13. Rinse 2 x with 1X Wash Buffer for 2 minutes at RT
14. Pour away excess liquid.
15. Pipette 240 µl from the Fast RED A-B on each slide.
16. Incubate for 10 minutes at RT.
17. Rinse 2-3 times with diH_2O.

HE - Staining

Materials: 200 ml counterstaining reagent (diluted 1:1, mix 100 ml Hematoxylin Gill's I with 100 ml diH_2O), 0.02% ammonium hydroxide solution (mix 1.43 ml 1N ammonium hydroxide with 250 ml diH_2O), 200 ml xylene, Gabis hot plate, Eukitt™ Covering medium, 24 mm x 50 mm cover glasses.

1. 2 minutes in hematoxylin staining solution.
2. Rinse with tap water 2-3 times.
3. In 0.02% ammonium hydroxide solution.
4. Rinse with tap water 2-3 times.
5. Place on the heating plate for 15 minutes at 60°C
6. place in Xylene until the barrier is dissolved
7. Place 1 drop Eukitt™ flashing medium and 24 mm x 50 mm cover glass.
8. Air dry for 5 minutes.

**Tissue Type**	**Pathology**	**RNAscope Target Retrieval Reagent**	**RNAscope Protease Plus**
Intenstine	Normal	15 Min	30 Min
Instenstine	Tumor	15 Min	30 Min
Embryo	Normal	15 Min	30 Min
Brain	Normal	15 Min	30 Min
Brain	Cancer	15 Min	30 Min

Spleen	Normal	15 Min	30 Min
Eye / Retina	Normal	15 Min	30 Min
Heart	Normal	15 Min	30 Min
Muscels	Normal	15 Min	30 Min
Vessels	Normal	15 Min	30 Min
Liver	Normal	30 Min	30 Min
Liver	Cancer	15 Min	30 Min
Kidney	Normal	15 Min	30 Min
Breast	Cancer	15 Min	30 Min
Breast(TMA)	Normal	15 Min	30 Min
Colon	Normal	15 Min	30 Min
Colon	Cancer	15 Min	30 Min
Lung	Normal	15 MIN	30 Min
Lung	Cancer	15 Min	30 Min
Prostata	Normal	15 Min	30 Min
Prostata	Cancer	15 Min	30 Min
Lymph node	Normal	15 Min	30 Min
Lymph nide	Cancer	15 Min	30 Min
Pancreas	Normal	15 Min	30 Min
Cervical	Normal	15 Min	30 Min
Cervical	Cancer	15 Min	30 Min
Cervical Dysplasia	Abnormal	15Min	30 Min
Head	Cancer	15 Min	30 Min
Neck	Cancer	15 Min	30 Min
Skin	Normal	15 Min	30 Min
Skin (TMA)	Normal	15 Min	30 Min
Melanoma	Normal	15 Min	30 Min
Melanoma (TMA)	Normal	15 Min	30 Min
Nervus	Cancer	15 Min	30 Min
Nervus (TMA)	Cancer	15 Min	30 Min
Placenta	Normal	15 Min	30 Min
Stomach (TMA)	Normal	15 Min	30 Min
Stomach (TMA)	Cancer	15 Min	30 Min
Cell Pellets, fixes 10% NBF	-	15 Min	30 Min
HeLa Zellen, fixed mit 10% Formaldehyde/PBS/ACD Control	-	15 Min	30 Min
