# Supplementary material for: Viral mapping in COVID-19 deceased in the Augsburg autopsy series of the first wave: A multiorgan and multimethodological approach
Source: PLoS One. 2021 Jul 19;16(7):e0254872. doi: 10.1371/journal.pone.0254872 (PMC8289110; doi:10.1371/journal.pone.0254872)
Supplement: S3 File — (PDF) [file pone.0254872.s003.pdf]

Protocol for SARS-CoV-2 Diagnostics:

SARS-CoV-2 is classified as a risk group 3 pathogen according to the WHO. Thus, potentially contaminated samples must be processed in an S2 laboratory. All samples are opened and pipetted under a class II safety cabinet. After lysis and purification of RNA, there is no risk of infection.

Basically, lysis is initiated in the S2 laboratory of the central laboratory according to protocol 57 (Promega):

1. Sample preparation:

1. Prepare 400 µl of water in a preparation tube.
2. Dip the swab into the water provided, squeeze it out and break or cut off the rod.
3. Close the preparation tube tightly and vortex briefly.

2. Extraction (central laboratory):

1. Pipette 300 µl Lysis Buffer (provided by Molecular Pathology), extraction control (10 µl) included in the detection kit, if applicable, in the indicated volume and 300 µl sample into a 1.5 or 2.0 ml Eppendorf reaction tube.
2. Close the lid.
3. Mixing by converting.
4. Label specimens accordingly.
5. Specimens are transferred to Molecular Pathology. This should be done as promptly as possible.

3. Extraction (Molecularpathology):

The extraction of the virus RNA is performed using the LEV blood DNA Kit AS1290. For the following steps, the Maxwell extraction robot (Promega) is set up under a class II safety cabinet to ensure appropriate occupational safety.

In addition, a mouth guard, gloves and disposable clothing must be worn during specimen processing. These are disposed of in a black garbage can after completion of the extraction and before leaving the room.

Empty lysate vessels are disposed of sealed in a table trash. The cartridges are likewise carefully disposed of in the table trash after extraction (The individual items cannot be sealed, the liquids they contain can be potentially infectious, please handle with care. After completion of the work, this table waste is disposed of in a black garbage can.

1. Place an appropriately required number of LEV cartridges in the sample rack and remove the protective foil.
2. Place one of the elution tubes included in the kit in the sample rack and fill it with 60 µl of the elution buffer provided. Make sure that the elution tubes are labeled according to the sample labeling from the central laboratory.
3. Place a plunger in the appropriate position in the cartridge.
4. Then transfer 600 µl of the lysate (from the central laboratory) to the first chamber.
5. Transfer the sample rack to the rack holder of the Maxwell.
6. In the device menu for LEV, select the program: DNA Blood and start the run.
7. After extraction, use the eluate according to the requirements of the subsequent test system.
8. After the end of the run, UV disinfection is performed on the Maxwell.
9. After completion of the extraction, the safety cabinet is closed and UV disinfection is also performed here (see UV disinfection safety cabinet).

4. UV disinfection safety cabinet:

1. Carry out a wipe disinfection. To do this, apply a disinfectant solution using a spray bottle and then wipe the surfaces with a cloth. Please dispose of the wipes in a black garbage can.
2. For UV disinfection, please lower the front window completely. To do this, press the close front window button on the touchpad until the window is closed.
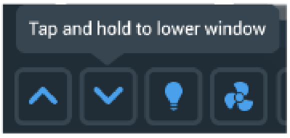

3. Press the UV button on the touchpad.
4. Wait until the UV disinfection cycle is complete.
5. The workbench is ready again for another extraction.

5. SARS-CoV-2 Assay (Singleplex):

After extraction of the nucleic acids, the test material is no longer to be treated as infectious. Nevertheless, the appropriate laboratory work instructions apply, such as the wearing of gloves once.

1. !!!ALL ASSAY components please THAW ON ICE and LEAVE IT THERE!!!
2. Prepare the master mix according to the Corona Assay Singleplex worklist under DNA workbench "Mastermix".
3. Add the extracted RNA accordingly.
4. Run on the QuantStudio5 Dx.

6. SARS-CoV-2 Assay (Multiplex):

Preparation of RT PCR:

1. Thaw reagents and the samples on ice.
2. Mix well by vortexing and centrifuge off.
3. Preparation of the positive control.
    a. Stock: 10,000 copies/µl 25 copies/µl
    b. Pipette 98 µl of COVID-19 control buffer into a 1.5 µl eppi and add 2 µl of COVID-19 control.
    c. Mixing and centrifugation.
    d. Pipette 87.5 µl of the COVID-19 control buffer into another 1.5 µl eppi and add 12.5 µl from the dilution in step 3b.
    e. Mixing and centrifugation.
4. Prepare the master mix according to the Corona Assay Multiplex Assay worklist under DNA Workbench "Master Mix".
5. Pipette according to the protocol onto the 96 well plate and add the controls and samples under the DNA workbench "DNA and RNA".
6. Close the plate with a foil and centrifuge off.
7. Start run on the QuantStudio5 Dx.

SARS-CoV-2 Multiplex Assay (TaqPath COVID-19 Thermo Fisher)

TaqPath™ 1-StepMultiplex MasterMix (No ROX™) (4X)
TaqPath™COVID-19 AssayMultiplex
TaqPath™ COVID-19 Control
MS2 Phage Control

Durchgeführt von (Mitarbeiterkürzel): SD

**TaqPath Covid 19**		Quantity	3	
**Part**	**Stock**	**final conc**	**1x**	**MM**
H20	-	-	12,50	41,25
4x MM	4x	1x	6,25	20,625
COV19 Assay	-	-	1,25	4,125
			20,00	
**Template RNA ‼**			**5,00**	

Temp	Time	Cycle
25°C	2'	
53°C	10'	
95°C	2'	
95°C	3"	
60°C	30"	40x

‼ **Positive Control: working solution 2 µl + 3 µl H2O**
‼ **Negative Control:Purified Negative Control 5 µl oder H2O 5 µl**
‼ **NTC: H_2O 5 µl**
